# Diffuse alveolar hemorrhage as a clinical manifestation of cardiac angiosarcoma in a pregnant woman with antiphospholipid syndrome: case report and review of the literature

**DOI:** 10.3332/ecancer.2025.1881

**Published:** 2025-04-01

**Authors:** R Hermosilla, S Martínez, A Perez, E López, R Granell

**Affiliations:** 1 Department of Gynecology and Obstetrics, Hospital Juan Ramón Jiménez, Huelva 21005, Spain; 2 Department of Anatomic Pathology, Hospital Juan Ramón Jiménez, Huelva 21005, Spain

**Keywords:** pregnancy, pericardial, tamponade, primary, cardiac, angiosarcoma, antiphospholipid syndrome

## Abstract

Primary cardiac angiosarcoma is a very rare and fast-growing tumour, where the coincidence of pregnancy and primary cardiac angiosarcoma is extremely rare. This makes diagnosis difficult and sometimes late, resulting in a poor prognosis from the moment of detection. We present the case of a 38-year-old pregnant woman in the 16th week of gestation diagnosed with obstetric antiphospholipid syndrome who came to the emergency department with asthenia, dyspnea, tachycardia and hypotension. A transthoracic ultrasound was performed with a diagnosis of pericardial tamponade. She was admitted to the intensive care unit for extrinsic cardiogenic shock. Transesophageal echography was performed, showing a large variegated mass in the right atrium, raising the differential diagnosis between atrial thrombus and myocardial tumour. Elective cesarean section was performed at 22 weeks of gestation. Months later, the patient was readmitted with a very unfavorable clinical evolution, experiencing diffuse alveolar hemorrhage/vasculitis refractory to life support measures. It was agreed to limit the therapeutic effort while awaiting the anatomopathological report of intracardiac biposia, subsequently confirming the histological nature of cardiac angiosarcoma. This case report highlights its rarity, showing a non-specific clinical presentation, which directed us towards a thrombosis in the right atrium and the fatal prognosis of angiosarcoma, mainly related to the progression of the disease due to a late diagnosis.

## Introduction

Angiosarcoma is a malignant neoplasm derived from rapidly proliferating anaplastic endothelial cells. Primary cardiac tumours have an incidence between 0.1% and 0.3% (40 times less frequent than metastases), 25% are malignant and most of them angiosarcomas. The coincidence of pregnancy and primary cardiac angiosarcoma is extremely rare, so much so that the few published data on cardiac angiosarcoma in pregnancy are mainly limited to case reports and case series [1–4].

Most tumours (about 90%) are located in the right atrium, originating in the lateral wall; the second most frequent location is the left atrium, followed by the right ventricle and, finally, the left ventricle. Due to the preferential right-sided location, patients with angiosarcoma often present with symptoms of heart failure and superior vena cava syndrome [1, 3]. Cardiac angiosarcoma is characterised by rapid growth resulting in a mass effect, causing blood flow obstruction, local invasion and/or distal embolisation. The clinical findings are not specific, the most frequent symptom is dyspnea, but many others have been described, such as atypical chest pain, cough, lower limb edema, arrhythmias, syncope, hemoptysis due to diffuse pulmonary hemorrhage, recurrent pulmonary embolism, as well as constitutional symptoms. Extension to the pericardium is frequent and usually with the formation of large, hemorrhagic and recurrent effusions, with or without cardiac tamponade [5–7]. Angiosarcomas usually metastasize to the lungs, although lesions have also been identified in other organs, such as the liver, lymph nodes, brain, bone, spleen, spleen, adrenal, pleura, diaphragm, kidneys, thyroid, intestine and skin [6, 8].

Imaging tests are essential for the study of an intracardiac mass because of their high diagnostic accuracy, accessibility and low cost. Echocardiography has become the gold standard for the primary evaluation of cardiac neoplasia. It provides essential data on tumour location, size, mobility and contours, while providing a complete morphological and functional cardiac evaluation [1, 9].

Angiosarcoma is a highly malignant tumour, mainly due to the relationship with metastases existing at the time of diagnosis, which explains the recurrence of the tumour after surgery, so it should be treated by a comprehensive approach that includes surgery, radiotherapy and chemotherapy [4, 9].

## Clinical case

The case is presented of a 38-year-old woman, pregnant at 16 weeks. Obstetric history: 4 previous pregnancies, 3 of them first trimester miscarriages and one term pregnancy that ended in cesarean section due to breech presentation. After repeated miscarriages, she was diagnosed with obstetric FAS with positive lupus anticoagulant antibodies, on treatment with acetylsalicylic acid (ASA) 100 mg/24 hour and heparin at a prophylactic dose. She consulted the emergency department for asthenia, dyspnea, tachycardia and hypotension, and transthoracic ultrasound was performed with a diagnosis of pericardial tamponade. She was admitted to the intensive care unit (ICU) for extrinsic cardiogenic shock, and 400 cc of hematic fluid was extracted by pericardiocentesis.

**Figure 1.**
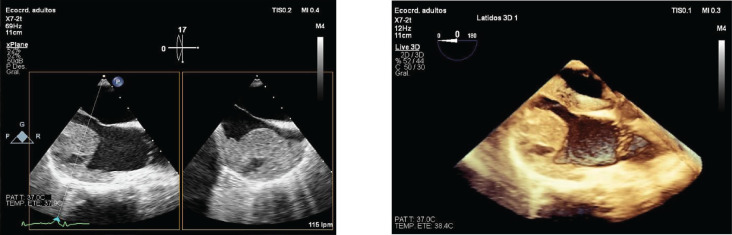
2D and 3D transesophageal echocardiography. Large mass protruding into the right atrium.

**Figure 2.**
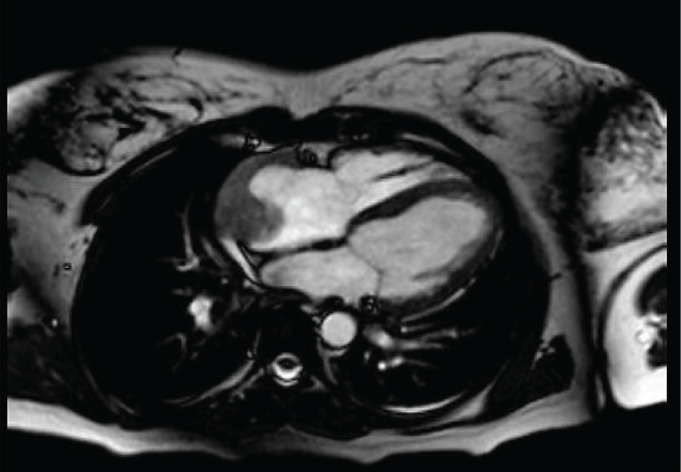
Thoracic magnetic resonance. Image compatible with intracardiac mass versus organized thrombus in right atrium.

Subsequently, a transesophageal ultrasound was performed, showing a large (43 × 55 × 35 mm) variegated, mobile and hyperechogenic mass, protruding into the right atrium, with a base of implantation in the right atrial appendage, compatible with both atrial myxoma and intracardiac thrombus (Figure 1), the latter being the main diagnosis of suspicion after performing a magnetic resonance imaging (Figure 2), being consequently treated as an organised thrombus. After stabilisation and adjustment of heparin to anticoagulant dose, the patient requested termination of pregnancy at 22 weeks, due to an acute anxious-depressive episode in the of the high maternal risk involved in continuing the pregnancy. A psychiatric opinion and the drafting of a medical report were necessary to support the legal termination of the pregnancy.

Routine ultrasound controls revealed total occlusive placenta previa, so a vaginal delivery was ruled out and it was decided to perform an elective cesarean section.

Corporal hysterotomy as well as bilateral tubal ligation at the patient’s express request, due to the high obstetric risk in future pregnancies, thus renouncing her genetic expectations. The patient was informed of the increased risk of acute pulmonary thromboembolism due to a possible detachment of the intraoperative thrombus, as well as the increased risk of thrombosis when discontinuing anticoagulant medication before and after surgery. The surgery was uneventful and the patient was discharged with ASA 100 mg/24 hour and Heparin 7,500 IU sc/24 hour.

The patient was readmitted 3 months later due to hemoptysis of several weeks of evolution, exertional dyspnea and febrile fever in the last 48 hours. Different complementary tests were performed, as follows: chest X-ray with bilateral interstitial infiltrate. Pulmonary CT angiography: multiple bilateral pulmonary consolidation foci, predominantly peripheral, surrounded by a reticular pattern with thickening of the intralobulbar septa and ground glass along with thrombus in the right atrium with moderate pericardial effusion (18 mm next to the right atrium). Given the patient’s underlying pathology, diffuse alveolar hemorrhage secondary to severe vasculitis/SAF was suspected. Fibrobronchoscopy: suggestive of diffuse alveolar hemorrhage.

Antibiotherapy and immunosuppressive treatment were started with intravenous boluses of methylprednisolone 500 mg/day for 4 days and cyclophosphamide (500 mg/m^2^ every 2 weeks). The patient was discharged after the stability of the picture, with Prednisone 50 mg/day for 1 month.

Subsequently, he was readmitted for persistent hemoptysis, fever and general malaise with progressive dyspnea and subscapular and thoracic pain, suggestive of thrombotic microangiopathy associated with primary PAS. During admission, a PET-CT scan was performed showing a central ametabolic lesion with peripheral hypercapillary halo in the right atrium, and pseudonodular lesions in both hypermetabolic lung fields, to rule out malignancy. Under these circumstances, an Eastern Cooperative Oncology Group (ECOG) score of 3 was performed.

The patient presented a torpid evolution with frank hemoptysis and respiratory worsening, requiring readmission to the ICU (ECOG 4). Intubation and mechanical ventilation were performed, requiring three sessions of plasmapheresis and administration of corticosteroids, pending histological report after endomyocardial biopsy.

**Figure 3.**
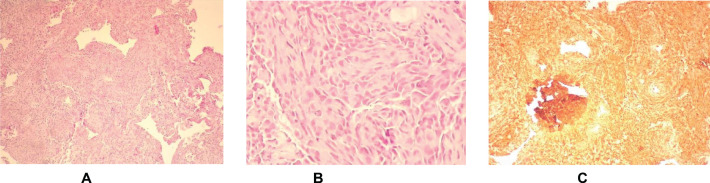
Histologic sections of cardiac angiosarcoma pathology. (a and b): Hematoxylin-eosin. Abundant spindleshaped pleomorphic cellularity with some atypical mitoses is observed. (c): Immunohistochemistry. Intense positivity for Vimentin supporting the mesenchymal origin of the neoplasm.

The pathology report described a poorly differentiated high-grade neoplasm compatible with cardiac angiosarcoma (Figure 3). Given the progression of the condition to severe respiratory failure refractory to the measures administered, it was decided to limit the therapeutic effort, and the patient died 7 months after the onset of the condition.

## Discussion

Primary cardiac angiosarcoma is a very scarcely documented neoplasm, with few cases described in pregnant women, which is why it is of interest due to its rarity. Angiosarcomas are fast growing, highly aggressive and frequently associated with the presence of metastases at the time of diagnosis (66% to 89%) [2, 7].

Angiosarcoma consists of a proliferation of endothelial cells that delimit irregular vascular spaces and anastomose together forming tumour masses, which invade the myocardium and pericardium, and present areas of necrosis and hemorrhage in their interior. This tumour affects mostly middle-aged men and can be located in any cardiac chamber or in the pericardium, with more than 80% being located intracavitary in the right atrium [1].

The most frequent symptom is dyspnea, but many other clinical findings have been described, such as atypical chest pain, cough, lower limb edema, arrhythmias, syncope, hemoptysis due to diffuse pulmonary hemorrhage, recurrent pulmonary embolism, constitutional symptoms and so on [6, 7]. That is why symptoms related to a cardiovascular etiology in a pregnant woman with autoimmune disease may point to vasculitis and/or catastrophic antiphospholipid syndrome and, as we have seen, it is important to consider this aggressive and rapidly fatal neoplasm in the differential diagnosis of intracardiac masses debuting with pericardial tamponade [1, 6, 7].

To carry out a good differential diagnosis, the use of complementary tests is necessary [8, 9]. Thanks to its high accuracy, accessibility and low cost, echocardiography is the best resource for the primary evaluation of a cardiac neoplasm. It provides essential data on the location, size, mobility and contours of the tumour, while providing a complete morphological and functional cardiac evaluation. The sensitivity of transthoracic and transoesophageal echocardiograms for detecting angiosarcomas are 93% and 97%, respectively. CT and MRI have a greater capacity to delimit and characterise these tumours [1, 10, 11]. However, in the present case, the primary cardiac angiosarcoma had a difficult and late diagnosis, being treated as an intracardiac thrombus given the similarity of the primary angiosarcoma to a thrombus.

Ultrastructure and taking into account the thrombogenic history of the patient due to the fact of having obstetric PAS. This is why we consider it appropriate to point out the importance of histological examination and immunohistochemical confirmation -with expression of vascular and endothelial antigens, including Factor VIII, CD31, CD34 and vascular endothelial growth factor VEGF- for an appropriate management, especially in case of accompanying manifestations such as tamponade or heart failure, as described by other authors [9–11].

In some cases of cardiac angiomyosarcoma, instead of using TNM, an additional classification is used, taking into account histologic grade and anatomic extension to establish staging and describe its malignity. According to the anatomopathological study in the case described, it was a poorly differentiated high-grade neoplasm, with rapid growth, greater aggressiveness and high probability of dissemination, especially to the lung and brain [11]; however, the existence of metastases could not be demonstrated by imaging tests.

Angiosarcoma is a highly malignant tumour, mainly due to the relationship with metastases existing at the time of diagnosis, which explains the recurrence of the tumour after surgery. Although new and promising therapies are being tested with selective drugs such as *tyrosine kinase inhibitors* and even immunotherapy, such as the *anti-PD-1 antibody* used with encouraging results in melanoma, it must be treated – in the absence of a standardised protocol – by means of a comprehensive therapeutic plan that includes surgery, radiotherapy and chemotherapy, and thus extend survival beyond 1 year. Treatment with radical surgery and adjuvant radiotherapy seems to be the most optimal combination, since chemotherapy in its various regimens (taxanes such as *paclitaxel*, anthracyclines such as *doxorubicin, liposome doxorubicin* plus *ifosfamide, doxorubicin* plus *the monoclonal antibody olaratumab*) is limited by comorbidities and the risk of related toxicity [11, 12].

Following a literature review in the Pubmed database, and as shown in the following table (Table 1), the course of this type of pregnancy-associated tumours is marked by severe complications, difficult therapeutic options and finally a rapid and fatal outcome.

## Conclusion

This clinical case highlights the need to consider malignancy in etiology of cardiac tamponade, given the ease with which some confounding factors can hinder and delay more specific therapeutic interventions.

## Conflicts of interest

The authors declare that there is no conflicts of interest.

## Funding

This work did not receive external funding.

## Figures and Tables

**Table 1.** Management after diagnosis of angiosarcoma.

	Age	Comorbidity	Symptoms	Metastasis	Management	Survival
Saraiva *et al* [1]	34 years old **32 sg**	HTA	Dyspnea and palpitations Heart failure	Liver	Urgent cesarean section Palliative surgery and chemotherapy six cycles of ifosfamide + epirubicin	>6 months asymptomatic and mass reduction. Survival unknown
Wang *et al* [4]	37 years **35 sg**	Unknown	Chest tightness Dyspnea	Pulmonary	Urgent cesarean section Tumor resection Adjuvant chemotherapy four cycles nabpaclitaxel+gemgitab ina RT+ IT: anlotinib	11 months after Q: recurrence in AD. Survival unknown
Waness *et al* [3]	30 years 38 sg	Asthma	Palpitations Chest pain Pericardial effusion	Hepatic Pulmonary	Pericardiocentesis Emergency caesarean section Adjuvant chemotherapy 3 paclitaxel cycles	Death after 3 months
Luo *et al* [5]	32 years old Nonpregnant	Unknown	Dyspnea	Pericardial infiltration Infiltration VC, Ao ascending, VT	Tumor resection Urgent cesarean section 27 sg	Survival unknown
Kudlicki et al [8]	33 years 23 sg	Unknown	Atrial fibrillation Dyspnea Tachycardia	Bone (rib, sternum, pelvis, vertebrae, vertebral	Adjuvant chemotherapy eight cycles ifosfamide+adriamycin +vincristine Spine Radiotherapy Pericardiocentesis Euthiocic labor 38 sg	Death at 13 months 6 months postsurgery
Azimi *et al* [6]	31 years 26 sg	None	Pulmonary edema Pericardial effusion	No Mtx	Postpartum detection AD tumor: tumor resection Chemotherapy four cycles placitaxel	Asymptomatic Survival unknown
Simon *et al* [2]	27 years 23 sg	None	Dyspnea Cardiac tamponade	No Mtx	Tumor resection Intrauterine fetal death Chemotherapy Adjuvant four cycles	8 months postsurgery tumor recurrence Cardiac transplantation Pulmonary Mtx Death at 20 months
Radulescu *et al* [9]	26 years old Nonpregnant	None	Chest tightness Dyspnea Palpitations	No Mtx	Tumor resection Chemotherapy adriamycin+dexamethaso ne+ifosfamide+citarabi na	6 months asymptomatic Survival unknown
Burns *et al* [10]	23 years 4 sg	None	Chest pain Tachycardia Cardiac tamponade Ascites	Liver	Pericardiocentesis ILE Chemotherapy two cycles of adriamycin+ifosfamide Gencitabine+docetaxel chemotherapy	9 months chemotherapy continues Survival unknown

HT arterial hypertension, sg weeks of gestation, Q surgery, AD right atrium, RT radiotherapy,

IT immunotherapy, VC vena cava, VT tricuspid valve, Ao aorta, Mtx metastasis, ILE legal interruption of pregnancy
